# An Updated Systematic Review and Meta-regression Analysis: Mental Disorders Among Adolescents in Juvenile Detention and Correctional Facilities

**DOI:** 10.1016/j.jaac.2020.01.015

**Published:** 2021-01

**Authors:** Gabrielle Beaudry, Rongqin Yu, Niklas Långström, Seena Fazel

**Affiliations:** a University of Oxford, UK; b Karolinska Institutet, Sweden

**Keywords:** criminal justice, detention, mental disorders, PTSD, systematic review

## Abstract

**Objective:**

To synthesize evidence on the prevalence of mental disorders in adolescents in juvenile detention and correctional facilities and examine sources of heterogeneity between studies.

**Method:**

Electronic databases and relevant reference lists were searched to identify surveys published from January 1966 to October 2019 that reported on the prevalence of mental disorders in unselected populations of detained adolescents. Data on the prevalence of a range of mental disorders (psychotic illnesses, major depression, attention-deficit/hyperactivity disorder [ADHD], conduct disorder, and posttraumatic stress disorder [PTSD]) along with predetermined study characteristics were extracted from the eligible studies. Analyses were reported separately for male and female adolescents, and findings were synthesized using random-effects models. Potential sources of heterogeneity were examined by meta-regression and subgroup analyses.

**Results:**

Forty-seven studies from 19 countries comprising 28,033 male and 4,754 female adolescents were identified. The mean age of adolescents assessed was 16 years (range, 10–19 years). In male adolescents, 2.7% (95% CI 2.0%–3.4%) had a diagnosis of psychotic illness; 10.1% (95% CI 8.1%–12.2%) major depression; 17.3% (95% CI 13.9%–20.7%) ADHD; 61.7% (95% CI 55.4%–67.9%) conduct disorder; and 8.6% (95% CI 6.4%–10.7%) PTSD. In female adolescents, 2.9% (95% CI 2.4%–3.5%) had a psychotic illness; 25.8% (95% CI 20.3%–31.3%) major depression; 17.5% (95% CI 12.1%–22.9%) ADHD; 59.0% (95% CI 44.9%–73.1%) conduct disorder; and 18.2% (95% CI 13.1%–23.2%) PTSD. Meta-regression found higher prevalences of ADHD and conduct disorder in investigations published after 2006. Female adolescents had higher prevalences of major depression and PTSD than male adolescents.

**Conclusion:**

Consideration should be given to reviewing whether health care services in juvenile detention can address these levels of psychiatric morbidity.

Adolescents account for approximately 5% of the custodial population in Western countries, and on any given day in the United States, 53,000 young people are detained in various correctional facilities.^1^ Psychiatric disorders are known to be prevalent in juvenile offenders.^2^ Furthermore, a number of studies indicate that psychiatric disorders in this population are linked to a wide range of negative outcomes, including elevated risk of repeat offenses,^3^^,^^4^ poor prognosis of mental health problems, high rates of substance misuse,^5^^,^^6^ increased likelihood to experience or perpetrate violence in intimate relationships, and psychosocial difficulties in adulthood.^7^

A previous systematic review and meta-analysis synthesized evidence up to 2006 on the prevalence of mental disorders in detained adolescents. The findings highlighted considerable mental health needs.^8^ Since then, a significant body of new primary research has been published. However, recent systematic reviews have been limited by their scope (eg, by including only English-language reports or not searching the gray literature), a lack of quantitative methods (including heterogeneity analyses), and the use of inconsistent time frames for psychiatric diagnoses (eg, in past month, past year, and lifetime).^9^, ^10^, ^11^ This article presents an updated systematic review and meta-analysis on the prevalence of mental disorders in detained adolescents, including posttraumatic stress disorder (PTSD),^12^ which has become increasingly researched in this population over the last decade. The findings should inform service provision, planning, and future research.

## Method

### Protocol and Registration

We conducted this systematic review following the Preferred Reporting Items for Systematic Review and Meta-Analyses^13^ and the Meta-analysis of Observational Studies in Epidemiology guidelines (see Table S1, available online).^14^ The study protocol was also registered with the PROSPERO International Prospective Register of Systematic Reviews (CRD42019117111).

### Search Strategy

**Figure 1:**
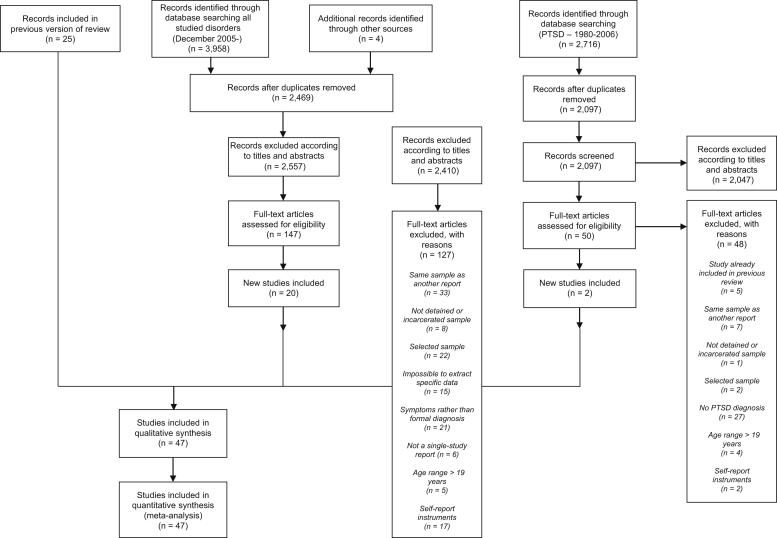
Flow Diagram Detailing the Search Strategy for the Updated Systematic Review (1966–2019) ***Note:****PTSD = posttraumatic stress disorder.*

We identified studies published between January 1966 and October 2019 reporting the prevalence of mental disorders in adolescents aged between 10 and 19 years in juvenile detention and correctional facilities. For the period January 1966 to May 2006, the methods were described in a previous review conducted by two of the authors (S.F. and N.L.).^8^ For this update, we searched the following electronic databases: EMBASE, PsycINFO, Medline, US National Criminal Justice Reference System Abstract Database, Global Health, and Google Scholar. Our search strategy featured terms related to adolescents (juvenile∗, adol∗, young∗, youth∗, boy∗, or girl∗) and custody (prison∗, jail∗, incarcerat∗, custod∗, imprison∗, or detain∗), which was identical to the previous review. For psychotic illnesses, major depression, attention-deficit/hyperactivity disorder (ADHD), and conduct disorder, new search dates ranged from December 2005 to October 2019. However, for PTSD, searches began in January 1980 to coincide with the addition of this disorder to *DSM-III*.^15^ Reference lists were hand-searched. No language restriction was set, and non-English surveys were translated (Figure 1).

### Study Eligibility

We included studies reporting diagnoses of psychotic illnesses, major depression, ADHD, conduct disorder, and PTSD among adolescents in juvenile detention and correctional facilities based on clinical examination or interviews conducted with semistructured diagnostic instruments.^16^ We defined adolescence from the age of 10 to 19 years,^17^ comparable with the previous review and consistent with research.^18^ We excluded studies that did not report the prevalence rates of mental disorders separately for male and female adolescents (with the exception of samples including <10% girls), surveys featuring enriched or selected samples of juveniles in custody, and studies that employed exclusively self-report instruments to diagnose individuals (but did include the Diagnostic Interview Schedule for Children [DISC], as it was typically administered in a semistructured way). Furthermore, included studies reported current prevalence of psychotic illnesses, major depression, ADHD, and PTSD or lifetime prevalence of conduct disorder that adhered to international classifications (*ICD* and *DSM*). Thus, one study^19^ was partially excluded because the prevalences of psychotic illnesses, major depression, and ADHD were reported for the past year rather than the past 6 months. Another reason to include PTSD was correspondence from the original review that recommended its inclusion to expand the clinical scope.^20^ For psychosis, we excluded one small study^21^ (n = 173) owing to being an outlier (11.0%).

### Data Extraction

One reviewer (G.B.) extracted data from the newly identified studies according to the protocol used in the previous review. In the case of any uncertainty in data extraction, R.Y. and S.F. were consulted. Gender-specific information was collected in regard to prespecified characteristics: geographic location, year of interview, sampling method (consecutive admissions, total population, random, stratified random, or some combination thereof), participation rate, number of interviewed adolescents, diagnostic instrument and criteria (*ICD* or *DSM*), type of interviewer (psychiatrist versus other), proportion of individuals diagnosed with each disorder, mean age and age range, mean duration of incarceration at the interview, and proportion with violent offenses.^8^ Authors of primary studies were contacted when further information was required (Table 1).

**Table 1:** Extracted Information From Included Samples, 1966–2019

Study	Country	Population	Type of custody	Sampling strategy	Proportion not consenting	Total number interviewed	Instrument	Diagnostic criteria	Diagnoses reported	Mean age (Years)	Age range (Years)	Interviewer	Time detained before interview	Proportion committed violent offenses
Bolton, 1976^52^	USA	Juvenile detention center	Not further specified	Stratified random	Not provided	502 boys 149 girls	Semistructured interview	*DSM-II*	PI	16	16–17	Layperson	4 days	Not provided
Chiles *et al.*, 1980^53^	USA	Juvenile detention center	Correctional	Consecutive (psychotic individuals excluded)	0%	94 boys 26 girls	Clinical	Research criteria of depression	MD	Not provided	13–15	Nonpsychiatrist	Up to 2 days	Not provided
Kashani *et al.*, 1980^60^	USA	Detention center	Evaluation and detention	Consecutive	Not provided	71 boys 29 girls	Clinical	*DSM-III*	MD	15	11–17	Psychiatrist	Mean 7 days	6%
Hollander and Turner, 1985^59^	USA	Convicted juvenile delinquents	Correctional	Consecutive	8%	185 boys	Clinical	*DSM-III*	PI ADHD	15	12–18	Staff psychologist and psychiatrist	Not provided	38%
Duclos *et al.*, 1998^57^	USA	Detention center	Not further specified	Consecutive	25%	86 boys 64 girls	DISC-2.3	*DSM-III-R*	MD ADHD CD PTSD	15	12–18	Nonpsychiatrist	Not provided	Not provided
Shelton, 1998^68^	USA	Detention facilities	Committal and detention facilities	Complete sample	8%	252 boys 60 girls	DISC	*DSM-III*	PI	16	12–18	Nonpsychiatrist	Not provided	Not provided
Ulzen *et al.*, 1998^70^	Canada	Detainees	Secure custodial facilities	Not provided	7%	38 boys 11 girls	DICA-R	*DSM-III-R*	MD ADHD CD PTSD	15	13–17	Research assistant	Not provided	Not provided
Atkins *et al.*, 1999^51^	USA	Central detention facility	Not further specified	Simple random	17%	71 boys 4 girls	DISC-2.3	DSM-III-R	ADHD CD	15	13–17	Social workers, nurses, medical students	Up to 6 months	Not provided
Lader *et al.*, 2000^62^	UK	Detainees	Local prison secure juvenile facility (Young Offender’s Institution)	Stratified random	2%	314 detainee and 169 sentenced boys 107 detained/sentenced girls	SCAN Clinical	*DSM-IV* *ICD-10* (MD)	PI MD Mania BP	Not provided	16–20	Psychiatrist	Modal categories 0–2 months, 6–11 months, and 0–2 months	19%
Nicol *et al.*, 2000^64^	UK	Detainees	Secure juvenile facility (Young Offender’s Institution)	Stratified random	Not provided	51 juveniles (estimate >90% boys)	K-SADS-E	*DSM-III-R*	PI MD	Not provided	13–17	Psychiatrist and nonpsychiatrist	Not provided	35%
Pliszka *et al.*, 2000^66^	USA	Juvenile detention center	Not further specified	Consecutive	0%	45 boys 5 girls	DISC-2.3	*DSM-III-R*	MD ADHD CD Mania BP	15	11–17	Nonpsychiatrist	Up to 4 days	Not provided
Robertson and Husain, 2001^28^	USA	Detention centers	Secure detention	Simple random	Not provided	168 boys 79 girls	APS JDI	*DSM-IV*	PI MD ADHD CD Mania	15	11–18	Mental health worker (nonpsychiatrist)	Mean 10.2 days	17% boys, 18% girls (self-report)
Dimond and Misch, 2002^55^	UK	Remand detainees	Secure juvenile facility (Young Offender’s Institution)	Consecutive	5%	19 boys	K-SADS-P	*DSM-IV*	PI MD CD BP	Not provided	15–16	Psychiatrist	Not provided	42%
Oliván Gonzalvo, 2002^65^	Spain	Juvenile detention center	Correctional	Consecutive	0%	35 girls	Clinical	*DSM-IV*	PI MD ADHD	15	14–17	Psychiatrist	Up to a few days	Not provided
Ruchkin *et al.*, 2002^67^	Russia	Juvenile detention center	Correctional	Complete sample	2%	370 boys	K-SADS-PL	*DSM-IV*	MD ADHD CD	16	14–19	Psychiatrist	Not provided	49%
Teplin *et al.*, 2002^69^	USA	Detainees in correctional facilities	Pretrial detention center	Stratified random	4%	1,172 boys 657 girls	DISC-2.3	*DSM-III-R*	PI MD ADHD CD Mania	15	10–18	Trained interviewer (Master’s in psychology or associated field)	Up to 2 days	Not provided
Waite and Neff, 2002^72^	USA	Juvenile detention center	Not further specified	Consecutive	0%	9,629 boys 1,190 girls	Clinical	*DSM-IV*	PI ADHD CD	16	11–18	Clinical psychologist	Up to a few days	18% (boys), 19% (girls)
Wasserman *et al.*, 2002^73^	USA	Reception for juvenile delinquents	Assessment before correctional placement	Simple random	3%	292 boys	Voice DISC-IV	*DSM-IV*	MD ADHD CD Mania PTSD	17	Not provided	Layperson	Mean 18.7 days	36%
Gosden *et al.*, 2003^58^	Denmark	Detainees	Prison and secure social services facility	Consecutive	21%	100 boys	SCAN	*ICD-10* *DSM-IV* (ADHD)	PI MD ADHD CD	17	15–17	Psychiatrist	Mean 11 days	86%
Abram *et al.*, 2004^12^	USA	Detainees in correctional facilities	Short-term detention	Stratified random	3%	532 boys 366 girls	DISC-IV	*DSM-IV*	PTSD	15	10–18	Trained interviewer (Master’s in psychology or associated field)	Up to 2 days	Not provided
Dixon *et al.*, 2004^56^	Australia	Juvenile detention center	For serious girl offenders	Consecutive	5%	100 girls	K-SADS-PL	*DSM-IV*	PI MD ADHD CD PTSD	16	13–19	Clinical psychologist	Not provided	71%
Lederman *et al.*, 2004^63^	USA	Juvenile detention	Before trial or long-term placement	Consecutive	27%	493 girls	DISC	*DSM-IV*	MD ADHD CD	15	10–17	Nonpsychiatrist	Up to 5 days	54%
Vreugdenhil *et al.*, 2004^71^	Netherlands	6 national detention centers	Not further specified	Consecutive	21%	204 boys	DISC-IV (DISC-2.3 for PI)	*DSM-IV* *DSM-III-R* (PI)	PI ADHD CD	16	12–18	Nonpsychiatrist	Mean 4 months	72%
Yoshinaga *et al.*, 2004^48^	Japan	Juvenile Classification Home	Short-term detention	Consecutive	0%	40 boys 8 girls	CAPS	*DSM-IV*	PTSD	17	14–19	Psychiatrist	Up to 4 weeks	Not provided
Abrantes *et al.*, 2005^50^	USA	2 juvenile detention centers	Not further specified	Consecutive	Not provided	218 boys 34 girls	PADDI	*DSM-IV*	PI MD CD Mania PTSD	16	13–18	Staff (nonpsychiatrist)	Not provided	27% (self-report)
Kuo *et al.*, 2005^61^	USA	Juvenile detention center	Secure placement	Consecutive	31%	36 boys 14 girls	Voice-DISC	*DSM-IV*	MD	Not provided	13–17	Nonpsychiatrist	Median 4 days	Not provided
Chitsabesan *et al.*, 2006^54^	UK	Detainees	Secure juvenile facility (Young Offender’s Institution)	Stratified random	7%	118 boys 33 girls	SNASA	*DSM-IV*	PI MD ADHD	16	13–18	Psychiatrist	Mean 4 months	Not provided
Hamerlynck *et al.*, 2007^39^	Netherlands	Detainees	3 juvenile justice institutions	Complete sample	7%	212 girls	K-SADS-P-L	*DSM-IV*	CD	16	12–19	Not provided	Up to 1 month	Not provided
Colins *et al.*, 2009^19^	Belgium	Detainees	3 youth detention centers	Simple random	15%	245 boys	DISC-IV	*DSM-IV*	CD PTSD	16	12–17	Trained interviewer (researcher and university students)	Between 3 days and 3 weeks	12%
Indig *et al.*, 2011^41^	Australia	Young people held in custody	8 juvenile detention centers and 1 juvenile correctional center	Simple random	5%	245 boys 39 girls	K-SADS-P-L	*DSM-IV*	PI MD ADHD CD PTSD	17	13–19	Trained juvenile justice psychologist	Not provided	Not reported for <19 years
Köhler *et al.*, 2009^43^	Germany	Prisoners on remand or in penal detention	Juvenile prison	Complete sample	7%	38 boys	SCID (German version)	*DSM-IV*	PI MD CD PTSD	Not provided	<18	Psychologist	Not provided	75% (not specific to <19 years)
Sørland and Kjelsberg, 2009^46^	Norway	Prisoners	Not further specified	Complete sample	5%	40 boys	K-SADS (Norwegian version)	*ICD-10*	MD CD	18	15–19	Researcher	60% during first 5 days of custody, 85% during first 18 days (range, 25–240 days)	Not provided
Karnik *et al.*, 2010^42^	USA	Detainees	Department of Corrections and Rehabilitation, Division of Juvenile Justice	Consecutive	1%	650 boys 140 girls	SCID (PI, MD, PTSD) DICA (ADHD) SIDP-IV (CD)	*DSM-IV*	PI MD ADHD CD PTSD	17	<16	Not provided	After 9 months	36%
Gretton and Clift, 2011^37^	Canada	Incarcerated youth	Provincial youth custody centers	Complete sample	Not provided	119 boys 54 girls	DISC-IV	*DSM-IV*	PI MD ADHD CD PTSD	16	13–18 (girls) 12–19 (boys)	Trained interviewer with advanced degrees in psychology	Not provided	83% (boys) 74% (girls)
Mitchell and Shaw, 2011^27^	UK	Remand and sentenced boys	Young Offender’s Institution	Simple random	7%	115 boys	K-SADS	*DSM-IV*	PI MD ADHD PTSD	17	15–17	Researcher with significant level of clinical experience	24 hours minimum	53%
Ghanizadeh *et al.*, 2012^36^	Iran	Incarcerated boys	Prison	Not provided	0%	100 boys	K-SADS (Farsi version)	*DSM-IV*	PI MD ADHD CD PTSD	17	12–19	Researcher	Not provided	83%
Harzke *et al.*, 2012^40^	USA	Youth entrants	Youth commission facilities	Complete sample	Not provided	10,469 boys 1,134 girls	Guided interview structure based on *DSM-IV*	*DSM-IV*	PI MD ADHD CD	Not provided	<19	Psychiatrists,clinical psychologists, associate psychologists, physicians, physician assistants, nurses	Up to 30 days	Assault (52.1%), sexual offenses (6.6%), murder/manslaughter (3.1%)^a^
Zhou *et al.*, 2012^47^	China	Detainees	2 youth detention centers	Complete sample	9%	232 boys	K-SADS-PL	*DSM-IV*	MD DP ADHD CD	17	15–17	Psychiatrist	Not provided	73%
Lennox *et al.*, 2013^44^	UK	Adolescent offenders	Young Offender’s Institution	Consecutive	3%	219 boys	K-SADS	*DSM-IV*	PI MD PTSD	17	15–18	Not provided	0–26 days	72%
Aida *et al.*, 2014^34^	Malaysia	Detainees	5 prisons that are designated centers for juvenile offenders	Simple random	0%	105 juveniles (estimate >90% boys)	MINI-KID	*DSM-IV* *ICD-10*	PI MD ADHD CD	17	14–17	Psychiatrist	Not provided	38%
Guebert and Olver, 2014^38^	Canada	Adolescents adjudicated under Youth Criminal Justice Act or former Young Offenders Act)	Not further specified	Not provided	Not provided	109 boys 77 girls	Diagnostic interview	*DSM-IV* or *DSM-IV-TR*	MD ADHD CD	16	Not provided	Pediatric psychiatrist, registered (usually doctoral level) psychologist	Not provided	83% (boys), 74% (girls)
Aebi *et al.*, 2015^33^	Austria	Male juvenile detainees	County jail	Consecutive	3%	259 boys	MINI-KID	*DSM-IV* *ICD-10*	ADHD PTSD	17	14–19	Psychiatry resident	Up to 4 days	8.5%
Dória *et al.*, 2015^35^	Brazil	Incarcerated boys	Socio-education center	Simple random	Not provided	69 boys	K-SADS-PL (Brazilian version)	*DSM-IV*	MD ADHD CD	16	12–16	Trained interviewer	15–30 days	Not provided
Lindblad *et al.*, 2015^45^	Russia	Incarcerated delinquents	Juvenile correctional center	Consecutive	2%	370 boys	K-SADS-PL	*DSM-IV*	PI ADHD CD PTSD	16	14–19	Child psychiatrist	Not provided	49%
Aebi *et al.*, 2016^32^	Switzerland	Detainees	Juvenile detention center	Consecutive	2%	158 boys	MINI-KID	*DSM-IV* *ICD-10*	ADHD CD PTSD	17	13–19	Psychiatrist, forensic psychologist	Not provided	63.9%
Kim *et al.*, 2017^21^	South Korea	Juvenile detainees	Male juvenile detention center	Consecutive	0%	173 boys	MINI K-SADS-PL (Korean version)	*DSM-IV* *ICD-10*	PI MD ADHD CD PTSD	18	15–19	Clinical psychologist	Not provided	60%
Schorr *et al.*, 2019^49^	Brazil	Juvenile offenders in temporary custody	Provisional detention center	Consecutive	0%	74 boys	Clinical	DSM-IV	CD	Not provided	15–17	Psychiatrist	Not provided	24% committed homicide offenses

Note: ADHD = attention-deficit/hyperactivity disorder; APS = Adolescent Psychopathology Scale; BP = bipolar disorder; CD = conduct disorder; DICA = Diagnostic Interview for Children and Adolescents (R = Revised); DISC = Diagnostic Interview Schedule for Children; JDI = Juvenile Detention Interview; K-SADS = Schedule for Affective Disorders and Schizophrenia for School Aged Children (P = Present, L = Lifetime, E = Epidemiologic); MD = major depression; MINI = Mini-International Neuropsychiatric Interview (KID = for Children and Adolescents); PADDI = Practical Adolescent Dual Diagnostic Interview; PI = psychotic illnesses; PTSD = posttraumatic stress disorder; SCAN = Schedules for Clinical Assessment in Neuropsychiatry; SCID = Structured Clinical Interview for DSM-IV Axis I, II and Personality; SIDP = Structured Interview for DSM-IV Personality; SNASA = Salford Needs Assessment Schedule for Adolescents.

a Percentages do not add up to 100%.

### Quality Assessment

Study quality was assessed in the included surveys using a modified version of the Newcastle-Ottawa Scale, which appraises sample representativeness and size, participation rate, statistical quality, and ascertainment of diagnosis.^22^^,^^23^ We employed the same version of the checklist used in a recent study of the prevalence of PTSD in prisoners.^24^ The potential total score ranged from 0 to 6 points. Studies with a score of 0 to 2 points were considered low quality, studies with scores of 3 to 4 points were considered medium quality, and studies with scores of 5 to 6 points were high quality (see Supplement 1 and Table S2, available online).

### Data Analysis

A random-effects meta-analysis was conducted to calculate pooled prevalence of each disorder, given that heterogeneity among studies was high.^25^ We aggregated smaller studies, for which the sample size was <100 individuals. For these small studies, prevalences reported in the text were from the nonaggregated data, whereas the figures were generated using results from the aggregated data. The Poisson distribution was used to obtain 95% confidence intervals when events were rare.^26^ Two studies^27^^,^^28^ for which the prevalence of psychotic illnesses was zero were imputed according to standard methods (ie, confidence intervals were calculated using “3” as the numerator and the real population size as the denominator).^29^ We reported the *I*^*2*^ statistic and Cochran's *Q* to indicate the degree of heterogeneity between studies. In line with guidelines, heterogeneity was considered to be low when *I*^*2*^ ranged from 0 to 40%; moderate, from 30% to 60%; substantial, from 50% to 90%; and considerable, from 75% to 100%.^30^ We conducted subgroup and meta-regression analyses to explore source of heterogeneity on a range of study characteristics: year of publication (≤2006 versus >2006), gender (male versus female), mean age (both as a continuous and as a dichotomous variable; ≤15 or >15 years), sample size (both as a continuous and as a dichotomous variable; ≤250 versus >250 adolescents), study origin (United States versus elsewhere), instrument (DISC versus other instruments), diagnostic criteria (*ICD* versus *DSM*), interviewer (psychiatrist versus nonpsychiatrist), sampling strategy (stratified/nonstratified random versus consecutive/complete) and study quality score (both as a continuous and as a dichotomous variable; high-quality studies versus low- and medium-quality studies)). We first conducted univariate meta-regression, followed by multivariable analysis including factors that reached statistical significance (set at *p* < .05) in the univariate models. To test group differences, subgroup analyses were conducted on all dichotomous variables. All analyses were performed using STATA statistical software package, version 13.0 using metan and metareg commands.^31^

## Results

**Figure 2:**
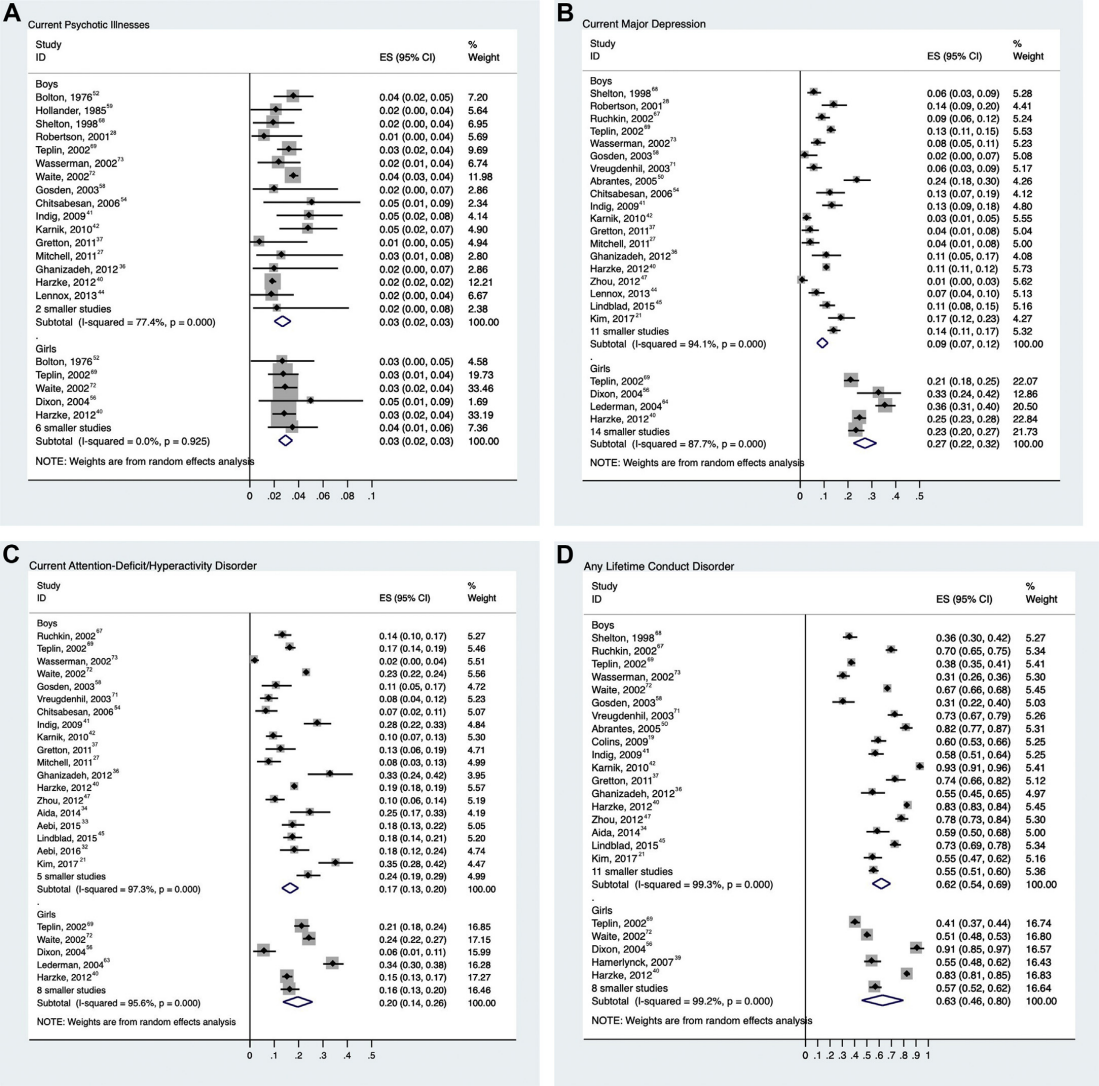

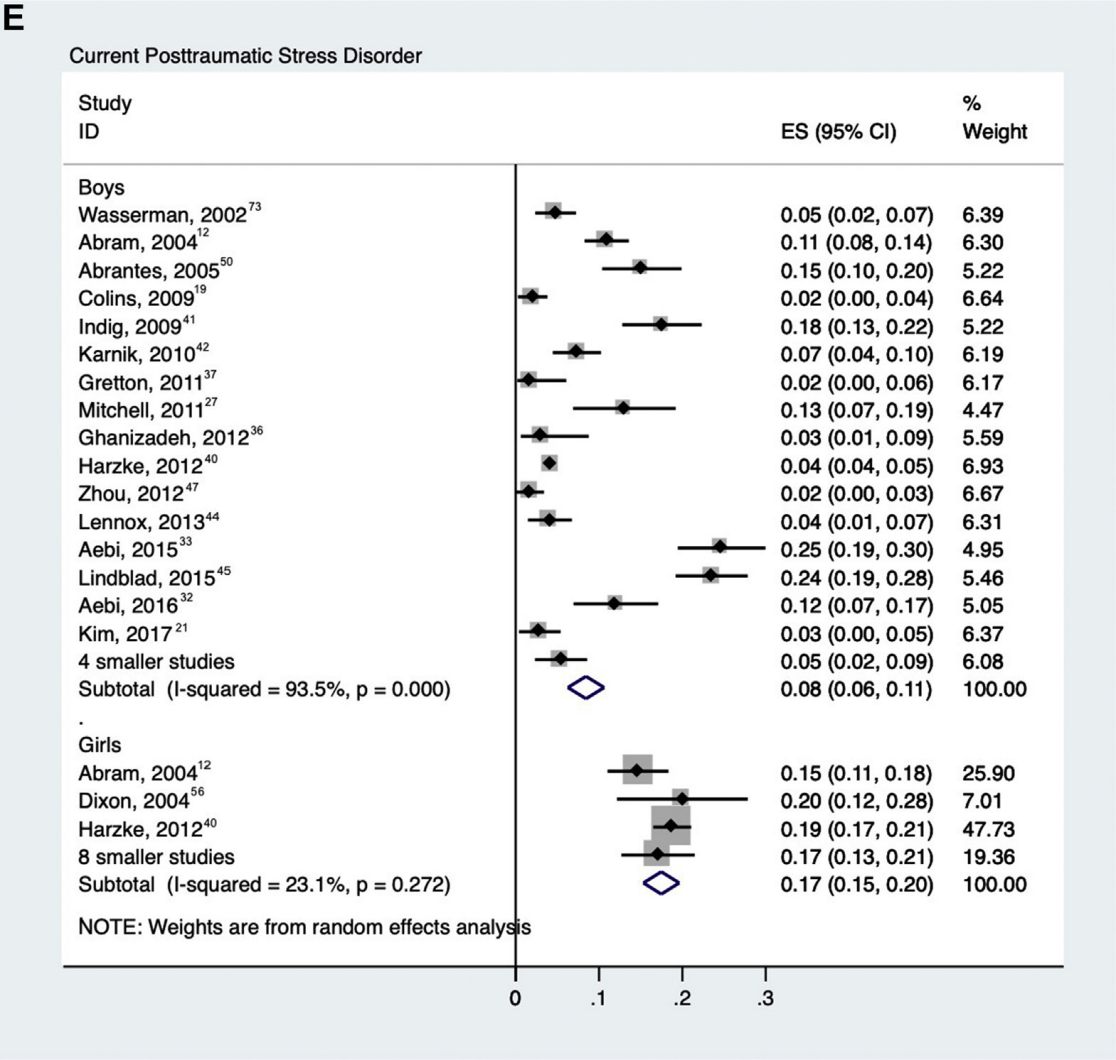
Prevalence of Specific Mental Disorders Among Incarcerated Male and Female Adolescents ***Note:****Error bars represent 95% CIs of prevalence. Smaller studies (n < 100) were aggregated. Subtotal is pooled prevalence estimate based on random effects models. ES = prevalence estimate.*

We identified 47 studies (46 different samples) from 19 different countries. Through our updated search, we found 22 new surveys.^12^^,^^19^^,^^21^^,^^27^^,^^32^, ^33^, ^34^, ^35^, ^36^, ^37^, ^38^, ^39^, ^40^, ^41^, ^42^, ^43^, ^44^, ^45^, ^46^, ^47^, ^48^, ^49^ We combined them with the 25 surveys identified in the previous review.^28^^,^^50^, ^51^, ^52^, ^53^, ^54^, ^55^, ^56^, ^57^, ^58^, ^59^, ^60^, ^61^, ^62^, ^63^, ^64^, ^65^, ^66^, ^67^, ^68^, ^69^, ^70^, ^71^, ^72^, ^73^ Two studies^12^^,^^69^ were based on the same sample, which provided data for different outcomes. The 47 studies included a total of 32,787 adolescents (28,033 male and 4,754 female [15%]) with mean age of 16 years (range, 10–19 years). Of studies, 18 were from the United States (n = 28,018, [86%])^12^^,^^28^^,^^40^^,^^42^^,^^50^, ^51^, ^52^, ^53^^,^^57^^,^^59^, ^60^, ^61^^,^^63^^,^^66^^,^^68^^,^^69^^,^^72^^,^^73^; six were from the United Kingdom (n = 1,145)^27^^,^^44^^,^^54^^,^^55^^,^^62^^,^^64^; three were from Canada (n = 408)^37^^,^^38^^,^^70^; two each were from Australia (n = 384),^41^^,^^56^ Brazil (n = 143),^35^^,^^49^ Russia (n = 740),^45^^,^^67^ and the Netherlands (n = 416)^39^^,^^71^; and one each was from Austria (n = 259),^33^ Belgium (n = 245),^19^ China (n = 232),^47^ Denmark (n = 100),^58^ Germany (n = 38),^43^ Iran (n = 100),^36^ Japan (n = 48),^48^ Malaysia (n = 105),^34^ Norway (n = 40),^46^ South Korea (n = 173),^21^ Spain (n = 35),^65^ and Switzerland (n = 158).^32^ These surveys were conducted using a range of sampling strategies, including consecutive recruitment of participants (n = 14,768),^21^^,^^32^^,^^33^^,^^42^^,^^44^^,^^45^^,^^48^, ^49^, ^50^^,^^53^^,^^55^, ^56^, ^57^, ^58^, ^59^, ^60^, ^61^^,^^63^^,^^65^^,^^66^^,^^71^^,^^72^ stratified random sampling (n = 3,272),^12^^,^^52^^,^^54^^,^^62^^,^^64^^,^^69^ simple random sampling (n = 1,432),^19^^,^^27^^,^^28^^,^^34^^,^^35^^,^^41^^,^^51^^,^^73^ and complete sampling (n = 12,980).^37^^,^^39^^,^^40^^,^^43^^,^^46^^,^^47^^,^^67^^,^^68^ Three studies (n = 335) did not report on their sampling method.^36^^,^^38^^,^^70^ Response rates were reported in 38 studies,^12^^,^^19^^,^^21^^,^^27^^,^^32^, ^33^, ^34^^,^^36^^,^^39^^,^^41^, ^42^, ^43^, ^44^, ^45^, ^46^, ^47^, ^48^, ^49^^,^^51^^,^^53^, ^54^, ^55^, ^56^, ^57^, ^58^, ^59^^,^^61^, ^62^, ^63^^,^^65^, ^66^, ^67^, ^68^, ^69^, ^70^, ^71^, ^72^, ^73^ and only seven of them (n = 1,317) reported rates ≤75%.^19^^,^^51^^,^^57^^,^^58^^,^^61^^,^^63^^,^^71^ Interviews were conducted using the following instruments: 12 used the Diagnostic Interview Schedule for Children and Adolescents,^12^^,^^19^^,^^37^^,^^51^^,^^57^^,^^61^^,^^63^^,^^66^^,^^68^^,^^69^^,^^71^^,^^73^ and 14 used the Schedule for Affective Disorders for School-Age Children, Present, Lifetime or Epidemiologic Version,^21^^,^^27^^,^^35^^,^^36^^,^^39^^,^^41^^,^^44^, ^45^, ^46^, ^47^^,^^55^^,^^56^^,^^64^^,^^67^ while the other surveys employed the Diagnostic Interview for Children and Adolescents,^42^^,^^70^ the Research Diagnostic Criteria for Depression,^53^ the Adolescent Psychopathology Scale and Juvenile Detention Interview,^28^ the Practical Adolescent Dual Diagnostic Interview,^50^ the Salford Needs Assessment Schedule for Adolescents,^54^ the Mini-International Neuropsychiatric Interview for Children and Adolescents,^32^, ^33^, ^34^ the Structured Clinical Interview for DSM-IV Axis I, II and Personality,^42^^,^^43^ the Clinician-Administered PTSD Scale from DSM-IV,^48^ or a semistructured interview.^52^ Most reported diagnoses were assigned using *DSM* criteria. However, one study provided *ICD-10* diagnoses,^46^ while others combined both *DSM* and *ICD-10* diagnoses.^21^^,^^32^, ^33^, ^34^^,^^58^^,^^62^ The diagnostic interviews were mostly conducted by psychiatrists,^33^^,^^34^^,^^41^^,^^45^^,^^47^, ^48^, ^49^^,^^54^^,^^55^^,^^58^^,^^60^^,^^62^^,^^65^^,^^67^ clinical psychologists,^21^^,^^43^^,^^56^^,^^72^ researchers and research assistants,^27^^,^^36^^,^^46^^,^^70^ or teams with diverse backgrounds.^19^^,^^28^^,^^32^^,^^35^^,^^37^^,^^38^^,^^40^^,^^50^^,^^51^^,^^59^^,^^64^ Most studies reported the types of offenses, and in accordance with previous research,^74^ we calculated the proportion of adolescents who committed violent offenses, which ranged from 6.0%^60^ to 86.0%.^58^ Figure 2 presents gender-specific prevalence estimates.

### Psychotic Illnesses

Prevalence of psychotic illness was reported in 21 studies, comprising 27,801 adolescents.^21^^,^^27^^,^^28^^,^^36^^,^^37^^,^^40^, ^41^, ^42^, ^43^, ^44^^,^^52^^,^^54^^,^^56^^,^^58^^,^^59^^,^^64^^,^^65^^,^^68^^,^^69^^,^^72^^,^^73^ Overall, 683 of 24,261 male adolescents were diagnosed with a current psychotic disorder (random-effects pooled prevalence 2.7%; 95% CI 2.0%–3.4%) (Figure 2a). There was substantial heterogeneity between surveys (*χ*^2^_17_ = 71, *p* < .001; *I*^*2*^ = 76%). Among female adolescents, 105 of 3,540 individuals were diagnosed with a current psychotic disorder (random-effects pooled prevalence 2.9%; 95% CI 2.4%–3.5%). Heterogeneity between studies was low (*χ*^2^_10_ = 5, *p* = .916; *I*^*2*^ = 0%). We found no associations between study characteristics and prevalence estimates in meta-regression.

### Major Depression

We identified 33 studies on major depression in 18,861 adolescents.^19^^,^^21^^,^^27^^,^^28^^,^^35^, ^36^, ^37^, ^38^^,^^40^, ^41^, ^42^, ^43^, ^44^, ^45^, ^46^, ^47^^,^^50^^,^^53^^,^^54^^,^^56^, ^57^, ^58^^,^^60^^,^^61^^,^^63^, ^64^, ^65^, ^66^, ^67^, ^68^, ^69^, ^70^, ^71^^,^^73^ Overall, 1,753 of 15,881 male adolescents (random-effects pooled prevalence 10.1%; 95% CI 8.1%–12.2%) (Figure 2b) and 774 of 2,980 female adolescents (25.8%; 95% CI 20.3%–31.3%) had a current major depression episode. There was considerable heterogeneity among both male (*χ*^2^_29_ = 339, *p* < .001; *I*^*2*^ = 91%) and female (*χ*^2^_17_ = 159, *p* < .001; *I*^*2*^ = 89%) samples. Meta-regression suggested that both gender and study quality were associated with heterogeneity among studies. Male adolescents (β = −.14, SE = .032; *p* < .001) and studies with higher quality scores (β = −.08, SE = .036; *p* = .040) reported lower prevalence.

### ADHD

We identified 27 articles^21^^,^^27^^,^^33^, ^34^, ^35^, ^36^, ^37^, ^38^^,^^40^, ^41^, ^42^^,^^45^^,^^47^^,^^54^^,^^56^, ^57^, ^58^^,^^63^^,^^65^, ^66^, ^67^^,^^69^, ^70^, ^71^, ^72^, ^73^ reporting on ADHD among 28,749 juveniles in custody. Overall, 4,951 of 24,824 male adolescents (random-effects pooled prevalence 17.3%; 95% CI 13.9%–20.7%) (Figure 2c) and 836 of 3,925 female adolescents were diagnosed with current ADHD (17.5%; 95% CI 12.1%–22.9%). Heterogeneity was high for male (*χ*^2^_23_ = 824, *p* < .001; *I*^*2*^ = 97%) and female (*χ*^2^_12_ = 179, *p* < .001; *I*^*2*^ = 93%) samples. Meta-regression found that heterogeneity was partly explained by the publication year (studies published after 2006 reporting a higher prevalence: β = .08, SE = .04; *p* = .03). In subgroup analyses, the pooled estimate of prevalence of studies published after 2006 was 20.4% (95% CI 17.4%–23.3%) compared with 13.6% (95% CI 8.4%–18.7%) before 2006.

### Conduct Disorder

We identified 31 studies on conduct disorder in 28,846 juveniles.^19^^,^^21^^,^^34^, ^35^, ^36^, ^37^, ^38^, ^39^, ^40^, ^41^, ^42^, ^43^^,^^45^, ^46^, ^47^^,^^49^, ^50^, ^51^^,^^55^, ^56^, ^57^, ^58^^,^^62^^,^^66^, ^67^, ^68^, ^69^, ^70^, ^71^, ^72^, ^73^ Overall, 18,042 of 25,184 male adolescents (random-effects pooled prevalence 61.7%; 95% CI 55.4%–67.9%) (Figure 2d) and 2,226 of 3,662 female adolescents (59.0%; 95% CI 44.9%–73.1%) had a diagnosis of any lifetime conduct disorder. Considerable heterogeneity was observed in male (*χ*^2^_28_ = 2,664, *p* < .001; *I*^*2*^ = 99%) and female (*χ*^2^_12_ = 1,127, *p* < .001; *I*^*2*^ = 99%) samples.

In meta-regression, studies published after 2006 (β = .19, SE = .07; *p* = .006) and studies with older participants (β = .12, SE = .05; *p* = .013) had higher prevalences. We also found lower prevalences of conduct disorder where the DISC was used (β = −.22, SE = .07; *p* = .004). None of these variables remained significant in multivariable meta-regression.

### PTSD

Twenty-one studies reported on PTSD^12^^,^^19^^,^^21^^,^^27^^,^^32^^,^^33^^,^^36^^,^^37^^,^^40^, ^41^, ^42^, ^43^, ^44^, ^45^^,^^47^^,^^48^^,^^50^^,^^56^^,^^57^^,^^70^^,^^73^ in 16,136 detained adolescents. Among 14,260 male adolescents, 832 (random-effects pooled prevalence 8.6%; 95% CI 6.4%–10.7%) were diagnosed with current PTSD (Figure 2e), and 334 of 1,876 female adolescents (18.2%; 95% CI 13.1%–23.2%) were diagnosed with current PTSD with substantial heterogeneity in male (*χ*^2^_19_ = 250, *p* < .001; *I*^*2*^ = 92%) and female (*χ*^2^_9_ = 41, *p* < .001; *I*^*2*^ = 78%) samples. Gender was the only factor associated with heterogeneity in meta-regression (male adolescents had a lower prevalence: β = −.10, SE = .04; *p* = .01).

### Heterogeneity Analyses

Table 2 presents the results from the meta-regression analyses assessing sample characteristics as possible sources of heterogeneity between studies. Influence analysis, which was performed by omitting one study at a time, reported no effect. Egger's regression test showed publication bias in surveys reporting prevalence of conduct disorder (*t* = −4.98, *p* = .03) and PTSD (*t* = 2.32, *p* = .02), both in male adolescents (see Figures S1–S5, available online).

**Table 2:** Univariate Meta-regression Analyses Examining Possible Sources of Between-Study Heterogeneity Among Adolescents in Juvenile Detention

Variable	Psychotic Illnesses			Major Depression			ADHD			Conduct Disorder			PTSD		
	β	SE	*p*	β	SE	*p*	β	SE	*p*	β	SE	*p*	β	SE	*p*
Year of publication: ≤2006 vs >2006	−.005	.004	.22	−.072	.037	.06	.081	.035	.028∗	.194	.066	.005∗	−.029	.039	.47
Gender: male vs female	−.004	.005	.42	−.144	.032	< .001∗	.002	.040	.96	.028	.079	.72	−.102	.037	.01∗
Mean age (continuous)	−.003	.004	.53	−.033	.024	.18	.003	.027	.91	.124	.047	.01	−.014	.027	.60
Mean age: ≤15 vs >15 years	−.005	.007	.46	−.048	.073	.52	−.022	.079	.79	.182	.163	.27	−.007	.050	.89
Study size (continuous)	.000	.000	.97	.000	.000	.69	.000	.000	.65	.000	.000	.38	.000	.000	.44
Study size: ≤250 vs >250 adolescents	.005	.005	.26	−.022	.045	.63	.002	.040	.96	−.001	.082	.99	.031	.038	.43
Study origin: USA vs elsewhere	.003	.005	.52	.044	.037	.25	−.029	.039	.46	−.094	.073	.21	−.016	.038	.67
Instrument: DISC vs other	−.005	.005	.33	−.051	.040	.21	−.057	.041	.17	−.218	.071	.004∗	−.071	.038	.07
Diagnostic criteria: *ICD* vs *DSM*	.006	.005	.20	.034	.074	.64	.008	.080	.92	−.123	.122	.32	−.050	.053	.36
Interviewer: psychiatrist vs nonpsychiatrist	−.006	.005	.19	−.050	.042	.25	−.012	.041	.78	.118	.073	.11	−.004	.045	.93
Sampling strategy: stratified/nonstratified vs consecutive/complete	−.003	.005	.53	−.021	.040	.60	−.010	.042	.81	.099	.080	.22	−.030	.039	.45
Study quality (continuous)	.003	.002	.17	−.029	.013	.04∗	.007	.018	.71	.048	.033	.16	−.004	.017	.81
Study quality: high-quality studies vs low- and medium-quality studies	.007	.004	.12	−.756	.036	.04∗	−.013	.041	.76	.044	.073	.55	−.003	.039	.93

Note: ADHD = attention-deficit/hyperactivity disorder; DISC = Diagnostic Interview Schedule for Children; PTSD = posttraumatic stress disorder.

∗ *p < .05*.

## Discussion

In this updated systematic review of the prevalence of mental disorders among adolescents in juvenile detention and correctional facilities, we identified 47 studies with 32,787 adolescents from 19 different countries. We doubled the number of primary studies compared with a 2008 systematic review.^8^ Moreover, we broadened our scope of search by adding a new psychiatric diagnosis (PTSD) and more carefully analyzed heterogeneity. The prevalence estimates confirm high levels of mental disorders in detained adolescents. The two commonest treatable disorders in male adolescents were depression (present in about 1 in 10) and ADHD (prevalent in 1 in 5). In female adolescents, approximately one in four had depression, and one in five had PTSD. We found higher prevalences of depression and PTSD in girls in custody compared with boys.

Our review suggests that mental disorders are substantially more common among detained adolescents compared with general population counterparts. Approximately 3% of detained adolescents were diagnosed with a current psychotic illness, a 10-fold increase compared with age-equivalent individuals in the general population.^75^^,^^76^ Higher prevalences of current major depression were found in both male (10%) and female (26%) adolescents compared with the general adolescent population (5% and 11%, respectively).^77^ About 1 out of 5 detained adolescents had ADHD compared to 1 out of 10 adolescents in the general population.^78^ Nearly two-thirds of detained adolescents were diagnosed with any lifetime conduct disorder, whereas the estimated lifetime rate of conduct disorder in US adolescents is approximately 10%.^79^ In addition, adolescents in detention also had higher rates of PTSD than those in the general population, 9% versus 2% in male adolescents and 18% versus 8% in female adolescents.^80^ These differences underscore the large burden of psychiatric morbidity in detained adolescents.

Apart from higher prevalence than the general population, prevalence estimates in adolescent juvenile detention and correctional facilities were also different from those found in adult prison populations. Psychotic illnesses and major depression appear to be more prevalent in adult prisoners than in adolescent custodial populations.^81^ However, the prevalence estimates for PTSD are similar in both groups.^24^ These comparisons suggest that the mental health needs of detained adolescents could be different from those of adult prisoners and may require separate and specifically targeted programs to meet these needs.

The prevalences for ADHD and conduct disorder are higher than in the previous 2008 review. Regarding ADHD, this upward trend may be specific to detained adolescents, as ADHD diagnoses in youths in the general population have not increased when standardized diagnostic methods are used.^82^ There are two possible explanations for this finding. First, increased prevalence could result from increased awareness of ADHD symptoms among health professionals working in custodial services. That is, the true prevalence of these disorders remains unchanged, but clinicians might be identifying them more accurately. Second, higher prevalence may result from improved identification of adolescents at high risk of reoffending over time. Some individuals with ADHD and conduct disorders who previously might not have been identified may be more likely to be selected for placement in custodial correctional facilities due to improved identification of these disorders.

Another main finding was the higher prevalence of major depression and PTSD in detained female adolescents compared with their male counterparts. These results are consistent with results from adult prison samples^24^^,^^81^^,^^83^ as well as the general population, military personnel, and terror attack survivors.^84^, ^85^, ^86^, ^87^ However, the explanations for this specific to incarcerated youths are not clear. Criminality in female adolescents may be more strongly associated with internalizing mental disorders than crime in male adolescents, or girls might be more vulnerable to adverse and traumatic experiences related to an antisocial lifestyle either within or outside the detention centers.

Finally, the funnel plot results suggest publication bias in male adolescents toward lower prevalence for conduct disorder and toward higher prevalence for PTSD. This could be due to the increased attention that trauma theory has received as a putative causal mechanism for juvenile criminality. In contrast, a highly prevalent descriptive diagnosis such as conduct disorder might be perceived as less useful for etiologic understanding, treatment planning, and primary prevention regarding juvenile delinquency.

One implication of this updated review is that there is no pressing need for conducting more primary prevalence studies, especially in high-income countries, considering that the evidence base is quite large and with most prevalence estimates remaining stable over time. Hence, future research could move toward treatment and interventions in custodial settings and investigate modifiable risk factors for adverse outcomes within custody such as self-harm and violence that may be associated with mental health problems. Effective treatment will likely improve prognosis and reduce suicidality, violence, and reoffending risk.^88^

Some limitations should be noted. First, owing to discrepancies in how substance use disorder and other mental disorders were classified between studies, it was not possible to reliably examine comorbidity. As adolescents who have comorbid disorders generally present an elevated criminogenic risk, future research on comorbidity is needed.^45^^,^^69^^,^^89^ Second, there were insufficient data on the type of facilities (pretrial versus sentenced; short-term versus long-term) where youths were detained. Therefore, we could not explore whether this variable was associated with heterogeneity. Future studies should consider reporting this information on juvenile justice facilities. Third, our analyses were solely based on formal diagnoses of mental disorders according to *DSM* and *ICD*, which provide standard ways of communication between mental health professionals. However, we did not report on subthreshold psychiatric symptoms, which future work could examine, as these individuals could benefit from preventive programs. An additional limitation from this review is that the quality appraisal scale was not specifically designed for the purpose of prison prevalence studies, and therefore some of the scoring made assumptions that need further examination (including a lower score for interviews conducted by laypersons using standardized measures versus unstructured clinical interviews conducted by psychiatrists or psychologists, although most of the latter also used standardized tools). Further, there were high levels of between-study heterogeneity. This is expected due to the differences in jurisdictions regarding whom they detain, availability and effectiveness of health care services, and prison environments. Therefore, further work could examine prevalence rates longitudinally in the same individuals to study trends over time. Moreover, we primarily used data from the US general population as a point of comparison for the calculated pooled prevalences because of similar diagnostic instruments, age ranges, and prevalence periods.^77^, ^78^, ^79^, ^80^ Nevertheless, as worldwide rates differ, including for ADHD between high-income countries, prevalences should be interpreted in relation to national or regional general population prevalences. Finally, it is notable that all included studies were conducted in high- and upper middle–income countries despite the global search. Determining whether new research in other countries is required will need to be balanced by information in this review, local needs, and whether such research can be linked to improved services.

In conclusion, our updated systematic review has reported high rates of treatable mental disorders in detained adolescents. The findings underscore the importance of access to mental health services and effective treatment. Such treatment will likely improve prognosis of this population, almost all of whom will reenter the community, and decrease risk of repeat offending, reducing the substantial social and financial costs related to imprisonment.^90^
